# Magnitude and determinants of unmet need for family planning among reproductive age women in East Africa: multilevel analysis of recent demographic and health survey data

**DOI:** 10.1186/s40834-022-00168-x

**Published:** 2022-01-25

**Authors:** Melsew Setegn Alie, Gossa Fetene Abebe, Yilkal Negesse

**Affiliations:** 1 Department of Reproductive health and Human nutrition, School of Public Health, College of Medicine and Health Science, Mizan-Tepi University, P.O.Box 260, Mizan-Aman, Ethiopia; 2 Department of Midwifery, College of Medicine and Health Science, Mizan-Tepi University, P.O.Box 260, Mizan-Aman, Ethiopia; 3 Department of Epidemiology and Biostatistics, School Public Health, College of Medicine and Health Science Mizan-Tepi University, P.O. Box 26o, Mizan-Aman, Ethiopia

**Keywords:** Unmet need, Family planning, Reproductive age women, East Africa

## Abstract

**Introduction:**

Unmet need for family planning is the main obstacle to achieve healthy timing and desired number of children. Decreasing the unmet need for FP respects and protects human right and help to decrease the influence on biodiversity. Unmet need for family planning is the contributor and devastating issue of maternal health. Therefore, meeting the unmet need of contraceptive averts the maternal death and poverty. Therefore, determining the magnitude and its determinants is very important to intervene and design appropriate program umbrella.

**Objective:**

To determine the magnitude and its determinants of unmet need for family planning among reproductive age women in East Africa.

**Method:**

This study was analyzed secondary data from Demographic and Health Surveys (DHS) of which contained detailed family planning for all interviewed women aged 15 to 49 years. The data were weighted using sampling weight before any statistical analysis to account the sampling design. STATA version 15 was used for extracting, editing, recoding, and multilevel analysis. Median odds ratio (MOR), proportional change in Variance (PCV), Intraclass correlation coefficient (ICC), and Akaike Information Criteria (AIC) was analyzed. Four model was build and the best model was selected based on the smallest Akaike Information Criteria (AIC). Both bivariable and multivariable multilevel analysis was done. Variable with *p*-value< 0.25 were selected for multivariable multilevel logistic regression analysis. Variables with p-value ≤5% declared as statistical significant with outcome variable.

**Results:**

The magnitude of unmet need for family planning was 24.66% (95%CI: 24.1–25.2). The identified determinants of unmet need for family planning was 30–39 years (AOR = 0.7; 95% CI 0.54–0.91), age of 40–49 (AOR = 0.76; 95% CI 0.58–0.99), rural residence (AOR = 1.17; 95% CI 1.02–1.34), female household head (AOR = 0.66; 95% CI 0.61–0.73), women having 4–6 child (AOR = 1.76; 95% CI 1.55–1.99), women having 7–9 child (AOR = 2.77; 95% CI 2.34–3.28) women having ≥10 child (AOR = 3.51; 95% CI 2.58–4.78), women who give their first birth 19-25 years (AOR = 1.1; 95% CI 1.0–1.26), 26–34 years (AOR = 1.4; 95% CI 1.19–1.83) ≥35 years (AOR = 2.1; 95% CI 1.1–4.27) and no fertility desire (AOR = 1.52; 95% CI 1.36–1.67) were the determinants of unmet need for family planning in east Africa.

**Conclusion:**

Unmet need in east Africa is high as compare to other previous study. Maternal age, residence, sex of household head, number of children, age at first birth and fertility desire were the determinants identified in this study. Therefore, health interventions that reduce unmet need which enhance family planning service delivery among rural, male-headed household, women having more than three children and women who had no fertility desire needed in advance. Policies and programs of unmet need should be tailored the rural, young and no fertility desire women as well as male headed households.

## Introduction

Unmet need for family planning (FP) means those sexually active and fecund women who are not using family planning even though they report wanting to delay or not wanting any more children [1, 2]. Unmet need for family planning had regional variation and great variability among each countries with in the region [3]. Worldwide, the projected change for unmet need for family planning is little, from 142 million in 2015 to 143 million in 2030 because of the increment in demand for family planning and the number of married or in union women increased in sub-Saharan African country [4]. Huge gaps and persistently increased demand of family planning is the contributors for low contraceptive prevalence. In sub-saharan African countries the projected prevalence of unmet need for family planning is remains high in 2030 [1, 2].

Healthy timing and getting the desired number of children has multidimensional benefit [5, 6]. Meeting the unmet need for FP in very essential for the countries education, maternal and child health and economic development of the country and also work for lowering fertility [6–8]. Working on women who have unmet need for FP prevents unintended pregnancies, unplanned birth, unsafe abortion, infant death and maternal death [9]. Decreasing the unmet need for FP respects and protects human right and help to decrease the influence on biodiversity [10]. Despite those benefits of contraceptive, in sub Saharan African countries there is social norm still favors the large families [11]. Worldwide many women wants to avoid pregnancy so they need effective contraception [5]. Currently, over 0.2 billion women especially in the developing world told have desire to delay or prevent pregnancy, while not using modern family planning methods [8, 12]. Unmet need for FP in Sub-saharan African countries is high in contrast it falls in other regions of the world [13]. Globally 0.35 billion of couples unable to access or limited to modern family planning, this situation sever in sub-Saharan African countries [14].

Globally 190 million reproductive age women need to avoid pregnancy but not use any modern contraceptive [15]. In Africa one from five women had unmet need for family planning [16]. The magnitude of unmet need for different countries is different such as in Ethiopia 22% [17] Liberia 35.9% [18], Burundi 32.4% [19], Botswana 9.6% [20], Gambia 17.9% [21], Latin America and Caribbean 32% [22], West and middle Africa 51% [22], Alexandria, Egypt 16.28% [23], Aseer region in Saudi Arabia 32.6 [24]. Determinants identified in the previous literature were age of women [13, 19–21, 25–27], marital status and educational status of women [13, 19, 25, 28], religion [13, 20], region [19, 21], ethnicity [21], working status [13, 25, 29], residence [13, 19, 29], household wealth status [13, 19, 29], media exposure [13, 19, 20], decision-making on spending personal earnings [13], previous use of FP [26], age at first marriage [28], educational status of their partner [25, 28, 29], gender of household head [13], parity [13, 19–21, 25, 29], partner attitude towards family planning [20, 28], current menstrual status [28], discussion with partner about FP [18, 20, 25, 28], home visit by FP workers [13, 28], husbands desire other child [19, 21], experienced the death sons [19], number of household/family members and optimal number of children [21] were the determinant factors.

Unmet need for family planning is the contributor and devastating issue of maternal health. Meting the unmet need of contraceptive averts the maternal death [30]. Even though International Conference on Population and Development (ICPD+ 5) set goal to the total reduction of unmet need for family planning in 2015 but the prevalence and its hindering factors still exist [31]. Sustainable development Goal (SDG) ensure university access of family planning (FP) and specifically goal 3 and 5 set goal for health and wellbeing of all and promoting gender equality and empowering of women therefore universal access to reproductive health services is one concern to achieve it [32]. However, the demand of family planning is still high especially in Africa since the increasing of married women and women’s in union [4] and in reviewing of different literature the factors contributing to the unmet need is different in different countries so knowing the determinates for this specific region is important to intervene.

Meeting the unmet need for family planning has many significances like reducing unintended pregnancy, abortion and maternal and child death [33]. Unmet need for family planning is one factor for low improvement of maternal health and contribute to high population growth especially in African region. Hence, multinational study is important for its improvement and design countrywide intervention and design region wide programs. Therefore, determining the magnitude and its determinants of unmet need for family planning is very important to design appropriate program umbrella. Assessing the magnitude and determinants of unmet need improve the reproductive health programs of the region. Maternal and child health is highly influencing by unmet need for family planning. To intervene on unmet need for family knowing the magnitude and factors affecting it is prerequisite. In east Africa maternal and child, health needs great improving so understanding determinants of unmet need for family important to design appropriate intervention for appropriate population. Evaluating the magnitude and determinants of unmet need for family planning is important to assess the health system disparities and evaluate their performance on their role in the reduction of unmet need. The community stakeholders will understand how much unmet need had a burden on child and maternal health improvement. Study on unmet need for family planning help the policy makers to design appropriate policy for all sexually active women. Understanding the magnitude and factors on unmet need is important to distribute information and knowledge on family planning through different media. Understanding the magnitude and factors of unmet need for family planning is important to initiate for collaborative work with enter sectoral like education sector to reduce unintended and adolescent pregnancy. Understanding magnitude and determinants of unmet need is important to fill the gap of meeting the need for family planning, which is essential to dropdown the school drop out of adolescent and increase the schooling of them. Therefore, this study aimed to determine magnitude and its determinant factors of unmet need for family planning among reproductive age women in East Africa.

## Method

### Data source and population

This study was analyzed secondary data from the recent Demographic and Health Surveys which contained detailed family planning for all interviewed women aged 15 to 49 years which were conducted from 2011 to 2018 were our data source. Data were obtained and extracted from kid record (KR) file. DHS is collected by a stratified, multi-stage (cluster), random sampling design. The detailed method of data collection were accessed at DHS database. The source population were all sexually active and married or living in union women in survey period across the east African countries whereas the study populations were all sexually active and married or living in union women in the survey period in the selected Enumeration Areas (EA). Study included all childbearing age-women found in the selected clusters at least one night before data collection period. The study population was all sexually active childbearing age women during the survey period. Sexually inactive, infecund and sterilized women were excluded from the study population.

### Variables and measurement

#### Dependent variable

The outcome variable is unmet need for FP where it is composed of unmet need for spacing and unmet need for limiting. It is a binary variable which women who experience unmet need is coded as ‘1’ yes while not having unmet need ‘0’. Total unmet need is calculated from unmet for limiting and unmeet for spacing.

#### Independent variable

In this study, both the individual and community level variables were included. The independent variables were age, residence, wealth status, sex of household head, media exposure, age at first sex, age at first birth, total number of children, fertility preference, working status, place of delivery and delivery by cesearan section. From the most recent demographic and health survey datasets the dependent variables were, maternal age (15–19, 20–24, 25–29, 30–34, 35–39, 40–49), residence (urban, rural), maternal occupation (working, not working), sex of the household head (male, female), wealth status (poor, middle and richer), media exposure (yes, no), age at first sex (< 15, 16–25, 26–34. ≥35) age at first birth (≤18, 19–25, 26–34, ≥35), total number of children (≤3,4–6, 7–9, ≥10), fertility preference (yes, undecided, no),place of delivery (home, undecided, no) and delivery by cesearan section (yes, no) were considered as independent variables.

### Operational definition

#### Age of respondents

Current age of the mother recoded in to four categories with values of“0” for < 20, “1” for 20–24,“2” for 25–29,“3” for 30–36 and“4” for ≥35 years.

#### Working status

Women occupation was No “if women were housewife and didn’t working”, and Yes “If a woman were working, she might be self-employed or government employed”.

#### Wealth status

Categorized as; poor “if woman was in poorer and poorest household”, middle and rich “if woman was in richer and richest household”.

#### Media exposure

a composite variable of frequency of listening radio, watching television and reading newspaper, in which households were said to have media exposure “if they have exposed to either of listening radio or watching television or reading newspaper at least one a week” and no “if did not have exposure to all of the above media sources”.

#### Age at first sex

In the current study age at first sex was categorized as; ≤15 year,16-25 years, 26–34 year and ≥ 35 years.

#### Age at first birth

According this study age at first birth was categorized as ≤18 year, 19-25 years, 26–34 year and ≥ 35 years.

#### Sex of household head

The variable sex of household head was corded as male and female in the dataset and we used without change.

#### Fertility preferences

Categorized as have another child “Yes” undecided, “undecided”, no more need, sterilized, declared infecund and never had sex “no” for further analysis.

#### Total number of children

The total number of children in the household was categorized as ≤3 child, 4-6child, 7-9child, ≥10child.

#### Place of delivery

categorized as health facility if women delivered in any health facility, home if the women delivered in home and other if the women delivered in religion place and neighbors home.

#### Delivery of cesearan section

If the women delivered in by cesearan section categorized as “yes” and if the women not delivered in cesearan section categorized as “no”.

### Data analysis

The variables of the study were extracted from kid record (KR) file data set using STATA version 15. Before any analysis, the data were weighted using sampling weight to account the sampling design. Editing, coding and recoding was done by STATA. After the data were cleaned, categorized, coded and weighted, we explored the descriptive statistics by using the frequencies and percentages of data and presented by using tables. Intraclass Correlation Coefficient (ICC), proportional change in variance (PCV) and median odds ratio (MOR) were calculated for the appropriateness of multilevel logistic regression and checking the presence of clustering. We used the ICC value greater than 5% to consider a variation of unmet need prevalence across the cluster Significant clustering was found therefore multilevel logistic regression were more appropriate. Four models were built; null model (model 0) only dependent variable, model 1 dependent variable and community level factors, model 2 dependent variable and individual level variable and the final model (model 3) was dependent variable and both community level and individual level factors. The best model was selected by comparing the AIC level and the model with smallest Akaike Information Criteria (AIC) is the best-fitted model. Therefore, a model with a small Akaike Information Criteria (AIC) value was selected and all interpretations and inferences were made based on this model. After selecting the best-fitted model, bivariable and multivariable multilevel logistic regression was done to determine the determinants of unmet need for family planning in east Africa. Both bivariable and multivariable multilevel analysis was done. Variable in bivariable analysis with *p* value < 0.25 were selected in multivariable multilevel analysis. Variables with a p value< 0.25 at bivariable analysis were entered into the multivariable multilevel logistic regression model. Finally, *P* value ≤0.05 to declare statistically significant variables.

## Result

### Characteristics of the study population

The descriptive analysis of this study shows that nearly half (49.22%) of the study participants were in the age category of 20–29 years old. Majority (74.8%) of the reproductive age women in east Africa residing rural area. Concerning with the wealth of the women nearly half (44.91%) of them were poor wealth status. We also describe the household head in east Africa. Majority (77.42%) of the households were male headed. Regarding with media exposure, majority (63.88%) of the women had no media exposure (Table 1).

**Table 1:** Socio-demographic and economic distribution of the study participants in east Africa

Variables	Frequency	Percentage (%)
Maternal age		
15–19	1996	4.47
20–29	21,994	49.22
30–39	16,943	37.91
40–49	3754	8.40
Residence		
Urban	11,263	25.20
Rural	33,422	74.80
Maternal working status		
Working	25,704	57.52
Not working	18,981	42.48
Wealth status		
Poor	20,069	44.91
Middle	8960	20.05
Rich	15,656	35.04
Sex of household head		
Male	34,596	77.42
Female	10,089	22.58
Media exposure		
Yes	16,139	36.12
No	28,546	63.88

### Obstetric and reproductive health characteristics

Majority (70.79%) of the reproductive age women start their first sex at age of 16–25 years old. More than half (51.44%) of the reproductive age women give their first birth at 19–25 years old. Fifty three percent of the women had less than or equal to three child before the survey. Regarding to unmet need for limiting and spacing, it shows 15.28 and 9.37% of the women had unmet need for spacing and limiting respectively. The overall unmet need for family was 24.66% (Table 2).

**Table 2:** Obstetric and reproductive health characteristics of the study participants in east Africa

Variables	Frequency	Percentage (%)
Age at first sex		
≤ 15 years	12,045	26.96
16–25 years	31,630	70.79
26–34 years	961	2.15
≥ 35 years	49	0.11
Age at first birth		
≤ 18 years	18,442	41.27
19–25 years	22,985	51.44
26–34 years	3148	7.04
≥ 35 years	111	0.25
Total number of children		
≤ 3 child	23,702	53.04
4–6 child	14,423	32.28
7–9 child	5510	12.33
≥ 10 child	1050	2.35
Fertility preference		
Yes	25,372	56.78
Undecided	1776	3.97
No	17,537	39.25
Place of delivery		
Health facility	27,671	64.46
Home	14,337	33.40
Others	918	2.14
Delivery by cesearan section		
Yes	2495	5.59
No	42,0100	94.41
Unmet need for spacing		
Yes	6814	15.28
No	37,769	84.72
Unmet need for limiting		
Yes	4180	9.37
No	40,405	90.53
Total unmet need		
Yes	10,993	24.66
No	33,592	75.34

### Magnitude of unmet among reproductive age women in East Africa by different characteristics

As shown in Table 3, the prevalence of unmet need among reproductive age women in east Africa were 24.66% (95%CI: 24.1–25.2). Among rural residents, 25.26% of them had unmet need for family planning. Among age group 40–49 the prevalence of unmet need for family were 37.63%. Based on media exposure the higher prevalence shown in women with no media exposure (25.63%). Concerning to sex of the household head male headed household experienced high prevalence of unmet need (25.57%).The women who had more than or equal to 10 children ever were experienced highest unmet need for family planning (47.31%)(Table 3).

**Table 3:** Prevalence of unmet need for family planning by different characteristics among reproductive age women in east Africa

Variable	Category	Magnitude of unmet need in percent (%)
Maternal age	15–19	18.22
	20–29	19.80
	30–39	26.36
	40–49	37.63
Residence	Rural	25.26
	Urban	19.78
Working status	Working	26.89
	Not working	19.40
Wealth status	Poor	23.83
	Middle	25.73
	Rich	22.40
Husbands working status	Working	27.00
	Not working	24.38
Sex of household head	Male	25.57
	Female	18.02
Media exposure	Yes	20.40
	No	25.63
Age at first sex	≤15 years	24.78
	16–25 years	23.25
	26–34 years	23.61
	≥35 years	23.73
Age at first birth	≤18 years	23.63
	19–25 years	23.75
	26–34 years	23.07
	≥35 years	25.62
Total number of children	≤3 child	17.44
	4–6 child	27.41
	7-9child	37.87
	≥10 child	47.31
Fertility preference	Yes	47.21
	Undecided	4.24
	No	48.54
Place of delivery	Health facility	22.89
	Home	25.06
	Others	27.01
Delivery by cesearan section ever	Yes	18.37
	No	24.00
Overall prevalence	24.66	

### Random effect analysis

Multilevel analysis is necessary because there is a significant clustering of unmet need for family planning in this DHS data. The intraclass correlation coefficient (ICC) was 24.6% means 24.6% of the variability in magnitude of unmeet for family planning among reproductive age women were attributed to the clusters. The median odds ratio (MOR) value of the null model 1.62 also indicates the presence of variation in unmet need for family planning between clusters. It means if we randomly select households from different clusters, those households at the cluster with higher unmet need for FP had 1.62 times higher chance of having unmet need compared to their counter parts. As shown in Table 4, model 3 has the smallest Akaike Information Criteria (AIC =44,561.88) as compared to random intercept only model or null model (AIC = 48,757.57), model with only community-level factors (AIC = 44,582.14) and model with only individual-level factors (AIC = 44,577.95) (Table 4). In addition the proportional change in variance (PCV) increases from 7.7% (null model) to 19.2% (model 3), indicating that mode 3 best explains the variability of unmet need. Therefore, this model is the best-fitted model for the data because it has the smallest AIC as compared to the rest models. So interpretation and reports were made based on this model.

**Table 4:** Model comparison and random effect analysis result

Parameter	Null model 0	Model 1	Model 2	Model 3
ICC	24.6%	23.44%	22.21%	21.87%
Variance	0.26 [0.22,0.31]	0.24 [0.20,0.29]	0.23 [0.19,0.28]	0.21 [0.18,0.27]
MOR	1.62	1.59	1.56	1.54
PCV	Ref.	7.7%	11.5%	19.2%
Model fitness				
AIC	48,757.57	48,582.14	44,577.95	44,561.88

### Determinants of unmet need for family planning

In the final model (model 3) both individual and community-level factors added for multilevel analysis, of which maternal age, residence, media exposure, working status, sex of household head, age at first birth, fertility desire and total number of children were significantly associated with unmet need for family planning in east Africa (Table 5). The odds of unmet need for family planning among women age group of 30–39 years were 30%(AOR = 0.7; 95% CI 0.54–0.91) less likely as compared to age group of 15–19 year. Women’s in age group of 40–49 were 24%(AOR = 0.76; 95% CI 0.58–0.99) less likely experienced unmet need for family planning as compare to women’s age 15–19 years. Those women’s residing in rural area were 1.17 time (AOR = 1.17; 95% CI 1.02–1.34) more likely experienced unmet need for family planning as compared with women’s in urban area. Those households lead by female were 34% (AOR = 0.66; 95% CI 0.61–0.73) less likely experienced unmet need for family planning as compare to counterpart. The odds of unmet need for family planning was 1.76(AOR = 1.76; 95% CI 1.55–1.99), 2.77(AOR = 2.77; 95% CI 2.34–3.28) and 3.51(AOR = 3.51; 95% CI 2.58–4.78) times more likely among women who had 4–6 child, 7–9 child and ≥ 10 child as compare to the compare to the women’s having ≤3 children respectively. The odds of unmet need for family planning were 1.1(AOR = 1.1; 95% CI 1.0–1.26), 1.4 (AOR = 1.4; 95% CI 1.19–1.83) 2.1(AOR = 2.1; 95% CI 1.1–4.27) times more likely among women’s who give firth birth at age of 19-25 years, 26–34 years, ≥35 years as compared with women’s who give birth 15–19 years respectively. Media exposure of the women is the other determinants of unmet need for family planning among reproductive age women in east Africa. The odds of unmet need for family planning was 13% less (AOR = 0.87; 95% CI 0.78–0.97) likely among women who had exposure to media than the women who had not media exposure (Table 5).

**Table 5:** Determinants of unmet need among reproductive age women in east Africa

Variables	Category	Model 0 (Null)	Mode 1 AOR(95% CI)	Mode 2 AOR(95% CI)	Model 3 AOR(95% CI)
훽0-intercept*		0.3 [0.28,0.31]	0.14 [0.12,0.18]	0.13 [0.1,0.17]	0.13 [0.1,0.17]
Maternal age	15–19			1	1
	20–29			0.84 [0.68,1.04]	0.82 [0.67,1.03]
	30–39			0.71 [0.55,0.90]	0.7 [0.54,0.91]
	40–49			0.75 [0.57,0.99]	0.76 [0.58,0.99]
Residence	Rural		1.51 [1.35,1.69]		1.17 [1.02,1.34]
	Urban		1		1
Maternal working status	Working			1.4 [1.28,1.54]	1.39 [1.26,1.52]^*^
	Not working			1	1
Wealth status	Poor			1	
	Middle			1.04 [0.91,1.19]	1.05 [0.92,1.2]
	Rich			1.02 [0.91,1.13]	1.06 [0.95,1.19]
Sex of household head	Male			1	1
	Female			0.67 [0.60,0.74]	0.66 [0.61,0.73]^*^
Media exposure	Yes			0.85 [0.76,0.94]	0.87 [0.87,0.97]
	No			1	1
Age at first sex	≤15			1	1
	16–25			0.98 [0.88,1.1]	0.97 [0.86,1.0]
	26–34			0.86 [0.62,1.18]	0.83 [0.6,1.15]
	≥35			0.89 [0.41,1.90]	0.87 [0.4,1.85]
Age at first birth	≤18			1	1
	19–25			1.14 [1.03,2.02]	1.1 [1.0,1.26]
	26–34			1.45 [1.17,1.80]	1.4 [1.19,1.83]
	≥35			2.1 [1.04,4.88]	2.1 [1.1,4.27]
Total number of children	≤3child			1	1
	4–6 child			1.78 [1.6, 2.02]	1.76 [1.55,1.99]^*^
	7–9 child			2.82 [2.39, 3.39]	2.77 [2.34,3.28]^*^
	≥10 child			3.58 [2.64, 4.88]	3.51 [2.58,4.78]^*^
Fertility desire	Yes			1	1
	Undecided			1.38 [1.11,1.72]	1.4 [1.12,1.74]
	No			1.50 [1.40,1.66]	1.51 [1.36,1.67]^*^
Place of delivery	Health facility			0.95 [0.86,1.04]	0.96 [0.87,1.06]
	Others^b^			0.99 [0.81,1.21]	0.98 [0.8,1.21]
	Home			1	1
Delivery by C/S	Yes			0.84 [0.72,0.98]	0.85 [0.73,1.0]
	No			1	1

^*^p-value < 0.0001, ^b^ in neighbor home, religious place

## Discussion

Achieving the universal access to reproductive health services is the agenda of 2030 sustainable development goal [32]. To achieve the sustainable development goal solving the problem of unmet need for family planning is very essential. Therefore, this study aimed to determine the magnitude and determinants of unmet need for family planning among reproductive age women in east Africa based on recent DHS data. The magnitude of unmet need for family planning in this study was 24.66 [95%CI: 24.1–25.2]. This finding is lower than the finding in Latin America and Caribbean,32% and west and middle Africa,51% [22], Aseer region of Saudi Arabia, 32% [24] and Burundi, 32.4% [19]. This could be due to the women’s demand for family planning variation between regions and variation in family planning availability. The Latin America, Caribbean, west and middle Africa study was done before the beginning of sustainable development goal but the current study analysis is the most recent DHS data. In addition this could be due the policies and programs focus on increasing access of family planning everywhere [34]. Other possible reasons of this variation could be due to the SDG and FP2020 advocates acceptable and reliable modern family planning globally [35]. However this finding is higher than the study conducted in Ethiopia, 22% [17], Gambia, 17.9% [21] Alexandria, Egypt,16.28 [23]. This could be due to in Ethiopia the health extension program improves the access and acceptability of family planning in rural as well as urban area of the country [36]. Other possible reasons could be due to the increased in demand of family planning in Africa. In addition, it could be due to the involvement of multi-country and multicultural society in the current study may contribute to magnitude variation.

This study also identify determinants of unmet need for family planning among reproductive age women. The odds of unmet need for family planning among women’s age 30–39 and 40–49 were 30 and 24% less likely as compare the women’s age of 15–19 years old. This finding is consistent with the study finding conducted in Nigeria [13], Burundi [19], Botswana [20],Gambia [21], Zambia [27] and Tigray region Ethiopia [25]. This could be due to unmet need for family planning relatively high among young than older women [37]. This could be due to young women’s give less attention by society than older one [38]. In addition, this could be due decrease the demand of family planning during older age. The odds of unmet need for family planning were 1.17 more likely among rural residence women as compare to urban residence. This finding is comparable with the study finding in Nigeria [13], Burundi [19] and Pakistan [29] which indicated unmet need is more likely among rural residences. This could be due to the similar health care facility in the country as well as the similar family planning policies of the countries. The better extension of maternal health service improves the use of family planning among women [39].

The odds of unmet need for family planning were 34% less likely among female-headed households as compare to male-headed house. This finding is in line with study conducted in Nigeria [13]. This could be due to the empowerment of women and increased life decision which associated with maternal health service use among the women [40]. The other possible reasons is female headed household may increase resource gain and control. The odds unmet need and number of children positively related. The odds of unmet need for family planning was 1.76, 2.77 and 3.51 times more likely among women who had 4–6 children, 7–9 children and ≥ 10 children as compare to the compare to the women who had less or equal to 3 child respectively. This finding is consistence with the study conducted in Gambia [21]. This could be due to the increased the women’s exposure to family planning and getting more information on family planning. The odds of unmet need for family planning among women who give their first birth at age of 19–25 years, 26–34 years and ≥ 35 years were 1.1, 1.4 and 2.1 times more likely as compare to the women who give their first birth of less than 18 years respectively. This might be due increased self-esteem and self-confidence on demanding of family planning that could contributes increased unmet of the women. The odds of undecided on fertility desire and no fertility desire were 1.4 and 1.5 time more likely to have unmet need for family planning. This could be due desire of women may give the women to less demand of family planning. The women who had media exposure were 13% less likely to have unmet need for family planning. This consistent with the study finding on Nigeria [13],Burundi [19], and Botswana [20] which shows media exposure is the determinants unmet need for family planning. This could be due increased getting more choice of family and getting more information on family planning.

## Conclusion

One of the target of sustainable development goal is universal access of reproductive and sexual health services that directly contribute to the reduction of infant and maternal mortality and it has indirect benefit of reduction of poverty, hunger, increasing the involvement of education of women and empowering of girls. Therefore understanding unmet need for family planning is one means to intervene and facilitate for the achievement of those sustainable development goals. Unmet need for family planning contributes for high prevalent of unintended pregnancy which finally leads to unsafe abortion. On the other dimension, unmet need for family planning indirectly contributes for poverty, low level of schooling and gender inequality. Therefore understanding the magnitude and its determinants is very crucial to design appropriate intervention to reduce unmet need which indirectly it decrease the unintended and unsafe abortion which is risk for maternal mortality. In this study, the magnitude of unmet need for family planning in east Africa is high as compare the previous works. This implies cross-country intervention and region based family planning policies is needed to reduce unmet need and improve maternal health. Therefore, the countries policy maker and program designer should design and implement region level interventions. Determinants identified in this study was maternal age, residence, sex of household head, media exposure, age at first birth, total number of children and fertility desire. Therefore improving the unmet need for family planning by working on rural residence who have late first birth, more number of children, male-headed household and no media exposure. Policies and programs of unmet need should be tailored the rural, young women with no media exposed and no fertility desire women as well as male headed households. The policymakers and implementers should promote the utilization of family planning through mass media for reduction of unmet need. The government should also design a new approach to reduce the unmet need for family planning for male-headed households, the women who had work and the household who have more than four children including the different possibilities for women who experience first sex earlier than 15 years and women who give their first birth greater than 26 years. Future researchers had better assess the capacity and accessibility of the local health systems, the level of decentralized decision making to use family planning, common cultural practices, attitude, and perception of women towards family planning service utilization.

## Data Availability

The datasets analyzed during the current study are available from the DHS data set.

## Abbreviations

AOR: Adjusted odds ratio
AIC: Akaike Information Criteria
CI: Confidence interval
DHS: Demographic and health survey
FP: Family planning
ICC: Intraclass correlation coefficient
PCV: Proportional change in variance
MOR: Median odds ratio
